# Sexually dimorphic control of crossover distribution by the conserved ATPase PCH-2 in *C. elegans*

**DOI:** 10.17912/micropub.biology.001802

**Published:** 2025-08-25

**Authors:** Alberto Herrera, Bianca Pacheco, Bhumil Patel, Kealani Holland, Needhi Bhalla

**Affiliations:** 1 Department of Molecular Cell and Developmental Biology, University of California, Santa Cruz, Santa Cruz, California, United States

## Abstract

Meiotic crossover recombination is essential for accurate chromosome segregation and the creation of new allele combinations that drive natural selection during evolution. Thus, the number and distribution of crossovers is exquisitely controlled. We have shown that the pachytene checkpoint component and conserved AAA-ATPase PCH-2 controls crossover number and distribution during oogenesis in *C. elegans* . To test if PCH-2 has similar effects during spermatogenesis, we monitored recombination across a single chromosome in control and *pch-2* mutant males. Our results demonstrate that PCH-2 's effect on crossover distribution during spermatogenesis is different than we observed in oogenesis, exhibiting sexual dimorphism.

**Figure 1. PCH-2 localizes to meiotic chromosomes and controls the distribution of crossovers in the male germline:**
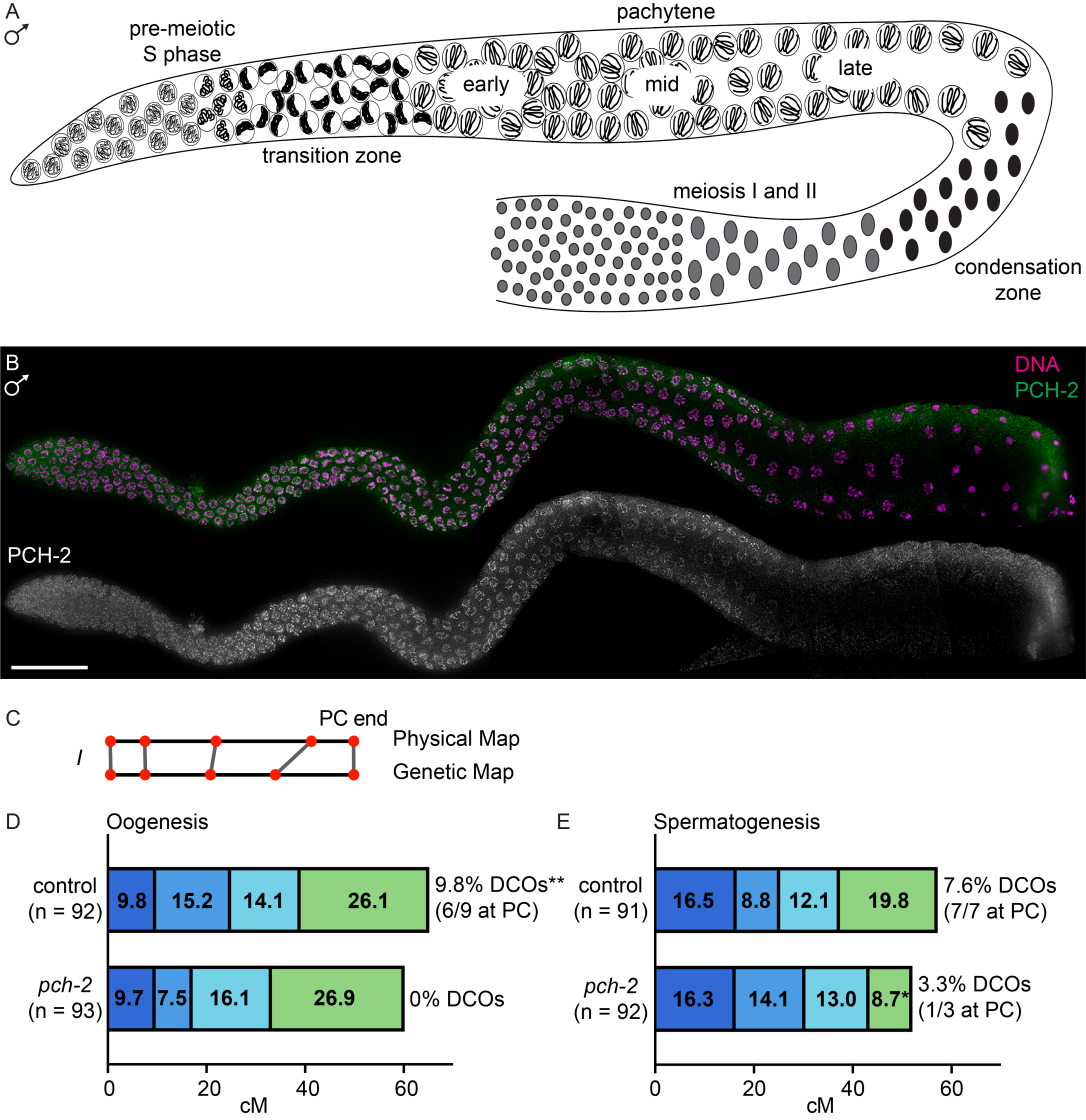
A. Cartoon of the male germline, labeled with stages of spermatogenesis. B. Image of male germline stained for PCH-2 (green and grayscale) and DNA (DAPI, magenta). Scale bar indicates 25 microns. C. Physical and genetic maps of Chromosome I are depicted to scale. D. Histogram showing genetic distances across Chromosome I during oogenesis in control animals and *pch-2* mutants. Data is from Patel et al., 2025. Genetic distance is shown in centimorgans (cM). E. Histogram showing genetic distance of the same four intervals during spermatogenesis in control animals and *pch-2* mutants. A * indicates a p-value < 0.05 and a ** indicates a p value < 0.01.

## Description

Meiosis is the specialized cell division that produces haploid gametes, such as sperm and eggs, for sexual reproduction. Despite sharing critical mechanistic similarities, meiosis also displays sexual dimorphism, where details like timing during development, duration of the cell division, length of meiotic chromosome axes, number and distribution of crossovers, and stringency of checkpoint activity varies between spermatogenesis (the production of sperm) and oogenesis (the production of eggs) (Cahoon and Libuda, 2019). Some of these dimorphisms can be observed in *C. elegans* , where meiotic prophase occurs 2.5-3 times faster in spermatogenesis than oogenesis (Jaramillo-Lambert et al., 2007) and male germlines do not exhibit germline apoptosis, either to maintain homeostasis (physiological apoptosis) (Gumienny et al., 1999) or in response to checkpoint activation (Jaramillo-Lambert et al., 2010).

A major goal of meiotic prophase is to establish a physical linkage between homologous chromosomes through the coordinated events of homolog pairing, synapsis and meiotic crossover recombination. This linkage, or chiasma, enables the accurate segregation of homologous chromosomes during meiosis I and introduces the genetic diversity that drives natural selection and evolution. Because of its importance, meiotic recombination is subject to striking levels of regulation to control crossover number and distribution, from its start (double strand break formation) to its end (crossover designation and resolution) (Gray and Cohen, 2016).

The regulation of meiotic recombination also shows sexual dimorphism (Cahoon and Libuda, 2019). In *C. elegans* , crossover recombination progresses faster during spermatogenesis than oogenesis (Cahoon et al., 2023a; Woglar and Villeneuve, 2018), reflecting either the acceleration of meiotic prophase or actual mechanistic differences. Whatever the reason, the observed differences in the progression of meiotic recombination also correlates with changes in crossover number, since double crossovers are observed both genetically and cytologically during spermatogenesis (Cahoon et al., 2023b; Gabdank and Fire, 2014; Lim et al., 2008). In oogenesis, we readily detect double crossovers genetically but not cytologically (Deshong et al., 2014; Patel et al., 2025).

We have shown that the conserved AAA-ATPase and pachytene checkpoint component, PCH-2 , controls the number and distribution of crossovers by inhibiting their formation throughout meiotic prophase during oogenesis in *C. elegans* (Deshong et al., 2014; Patel et al., 2025; Russo et al., 2023). Counterintuitively, this antagonism during oogenesis produces fewer crossovers in *pch-2* hermaphrodites (Deshong et al., 2014; Patel et al., 2025; Russo et al., 2023). Moreover, the remaining crossovers shift to the chromosome ends that undergo initial homolog pairing and synapsis and away from the gene-rich, central parts of the chromosome (Patel et al., 2025), potentially reflecting early homolog interactions and/or where double strand breaks happen in early prophase.

We favored this first hypothesis, that recombination shifted to sites of early homolog interactions in *pch-2* mutants. To test this hypothesis, we exploited the observations that pairing and synapsis are controlled by the same mechanisms during both oogenesis and spermatogenesis (Jaramillo-Lambert et al., 2010) but meiotic recombination displays sexual dimorphism (Cahoon et al., 2023a). We analyzed recombination in control and *pch-2* mutant males to determine what effect mutation of *pch-2* had on the distribution of crossovers during spermatogenesis. We found that changes in crossover distribution during spermatogenesis in *pch-2* mutants are not the same as observed in oogenesis, indicating that PCH-2 's effect on crossover number and distribution is sexually dimorphic. Moreover, the changes in crossover distribution we observe between oogenesis and spermatogenesis in *pch-2* mutants reflect mechanistic differences in the progression of meiotic prophase rather than evidence about early homolog interactions.

Before performing recombination analysis, we wanted to verify whether PCH-2 localized to meiotic chromosomes during spermatogenesis similar to what we have reported in hermaphrodites during oogenesis (Deshong et al., 2014). The male germline, like the hermaphrodite germline, is arranged in a spatio-temporal gradient: nuclei at the distal tip divide mitotically until they differentiate to go through pre-meiotic S phase. Entry into meiotic prophase is characterized by transition zone nuclei, corresponding to leptotene/zygotene, where chromosomes adopt a polarized morphology and undergo pairing and synapsis ( Figure 1A ). Fully synapsed chromosomes redisperse in pachytene, where the process of crossover recombination occur until crossovers are designated and cytologically marked in late pachytene ( Figure 1A ). Instead of diplotene/diakinesis, meiotic nuclei undergoing spermatogenesis condense before entering meiosis I and II ( Figure 1A ). PCH-2 forms foci in pre-meiotic S phase and the transition zone and localizes to meiotic chromosomes in pachytene ( Figure 1B ). PCH-2 persists on meiotic chromosomes until late pachytene, when it is removed where crossovers are cytologically marked. Thus, PCH-2 's localization to meiotic chromosomes is similar to what we observe in oogenesis in hermaphrodites.

To analyze recombination, we used wildtype Hawaiian CB4856 strain (HI) and Bristol N2 strains to assay recombination between single nucleotide polymorphisms (SNPs) that added or removed a restriction enzyme site (SNIP-SNP). We identified 5 SNPs that spanned 95% of Chromosome I ( Figure 1C ) and generated either control or *pch-2* mutant males that were heterozygous for N2 and Hawaiian SNPs (F1 animals). These heterozygous males were crossed to Hawaiian hermaphrodites that were wildtype or mutant for the *pch-2* gene and the F2 progeny were analyzed to monitor recombination in the heterozygous father.

In control males, we observed 7.6% double crossovers (DCOs in Figures 1D and 1E) on Chromosome I during spermatogenesis ( Figure 1E ), similar to what we had observed during oogenesis in control hermaphrodites ( Figure 1D and Patel et al., 2025). However, in contrast to what we observed on Chromosome I during oogenesis, 100% (7/7) of these double crossovers had one crossover that was at the end of the chromosome where pairing and synapsis initiate, also called the Pairing Center (PC) ( Figure 1E ) (MacQueen et al., 2005). Further, we saw an increase in recombination in the genetic interval at the non-PC end ( Figure 1E ), compared to what we observed during oogenesis ( Figure 1D ).

When we monitored recombination in *pch-2* mutant males, we observed a decrease in the frequency of double crossovers (3.3%) ( Figure 1E ) but not the significant decrease we observed during oogenesis ( Figure 1D ), consistent with the increase in cytological double crossovers during spermatogenesis. Moreover, 33% of double crossovers included one crossover at the PC end in *pch-2* mutant males, in contrast to the 100% we observed in control males ( Figure 1E ). While not a significant result, this suggests that mutation of *pch-2* does have subtle consequences for crossover formation at PCs. Finally, the most dramatic shift in recombination we could detect in *pch-2* mutant males was a significant reduction in recombination at the PC end and an increase in a more central, gene-rich, genetic interval ( Figure 1E ), the opposite of the phenotype we observed during oogenesis in *pch-2* mutants (Patel et al., 2025).

Since homolog pairing and synapsis also rely on Pairing Centers in males (Jaramillo-Lambert et al., 2010), these results do not support our hypothesis that PCH-2 specifically inhibits crossovers at regions of the chromosome that are likely to undergo early homolog interactions where pairing and synapsis initiate during oogenesis or spermatogenesis. Instead, these changes in crossover distribution in *pch-2* mutants likely reflect where double strand breaks happen early in meiotic prophase (Patel et al., 2025). These data also suggest that other factors, including the timing of meiotic prophase (Jaramillo-Lambert et al., 2007), the acceleration of meiotic recombination (Cahoon et al., 2023a), the absence of germline apoptosis (Gumienny et al., 1999) and/or the presence of the unpaired, unsynapsed X chromosome in the male germline that undergoes meiotic silencing (Maine, 2010), contribute to how PCH-2 controls the distribution of crossovers during spermatogenesis. Since we see variations in how PCH-2 controls crossover distribution between spermatogenesis and oogenesis, even in the same model system, these results also reinforce that the changes in crossover number and distribution observed in *pch-2* mutants across model systems likely reflect mechanistic differences that ensure proper regulation of crossover number and distribution that have been selected for in those systems (Bhalla, 2023).

## Methods

***C. elegans*** **strains and genetics:**

The Bristol N2 C. *elegans* strain (Brenner, 1974) was used as the wild-type control for all experiments. Strains were maintained on Nematode Growth Media seeded with OP50 bacteria and grown at 20°C under standard conditions for all experiments. The mutations and transgenes used in our experiments were:

LG II: *pch-2 ( tm1458 )*

LG V: *bcIs39 (Plin-15:: ced-1 ::GFP)*

**DAPI staining and Immunofluorescence:**

Adult males were fixed and stained 24-26 hours post L4 larval stage, similar to (Russo et al., 2023). For analyzing PCH-2 localization, rabbit anti- PCH-2 primary antibodies (Deshong et al., 2014) were used at 1:250 dilution and Alexa488 anti rabbit (Invitrogen) secondary antibodies were used at a 1:500 dilution.

Images of immunostaining experiments were obtained using a DeltaVision Personal DV system (Applied Precision) equipped with a 100x N.A. 1.40 oil-immersion objective (Olympus), resulting in an effective XY pixel spacing of 0.064 microns/pixel. Z-stacks were collected at 0.2-μm Z-spacing and processed by constrained, iterative deconvolution. Imaging, and image scaling were performed using functions in the softWoRx software package. Projections were generated using a maximum intensity algorithm and pseudo colored in Adobe Photoshop.

**Genetic analysis of Recombination**

The wildtype Hawaiian CB4856 strain (HI) and the Bristol N2 strain were used to assay recombination between single nucleotide polymorphisms (SNPs) on Chromosome I (Bazan and Hillers, 2011; Wicks et al., 2001). The SNPs, primers, enzymes used for restriction digests and expected fragment sizes are included in the chart below.

To measure wild-type recombination, N2 males containing *bcIs39* were crossed to Hawaiian CB4856 worms. Cross-progeny males were identified and contained one N2 and one CB4856 chromosome. These were assayed for recombination by crossing with CB4856 hermaphrodites. Cross-progeny hermaphrodites containing *bcIs39* from the resulting cross were isolated as L4s, and then cultured individually in 96-well plates in liquid S-media complete supplemented with HB101 . Four days after initial culturing, starved populations were lysed and used for PCR and restriction digest to detect N2 and CB4856 SNP alleles.

For recombination in *pch-2* mutants, strains homozygous for the CB4856 background of the relevant SNPs were created by backcrossing *pch-2* mutants to worms of the CB4856 background at least eight times and verifying the presence of Hawaiian SNPs on all chromosomes tested in the recombination assay. These Hawaiianized *pch-2* mutants were then mated with *pch-2 ; bcIs39* . Subsequent steps were performed as in the wildtype worms.

**Table:** 

SNP	Primer Name	Primer GeneticLocation	Primer Sequence FOR	Primer Sequence REV	Restriction enzyme	N2	HI
IA	F56C11	-19	ATGCCAGTGATAAGGAACGG	TCACATCCCTTGTCGATGAA	DraI	354, 146	500
IB	Y71G12	–12.3	GACAATGACCAATAAGACG	GATCCGTGAAATTGTTCCG	BsrI	440, 125	364, 125, 76
IC	K04F10	0.9	ATCATTCTCCAGGCCACGTTAC	CTGAACTAGTCGAACAAACCCC	NdeI	594	300, 294
ID	T07D10	13.6	CTTGGTGTGGGGAGAGTATAGG	TTTGTCCGGATTGACTCTGC	Sau3AI	303, 63	207, 96, 63
IE	ZK909	28.8	CACAAGTGGTTTGGAAGTACCG	CAACAAAGGGATAGATCACGGG	HindIII	450	236, 214

**Figures and Statistics:**

The figure was assembled using Adobe Illustrator. All histograms were generated using Prism Graphpad. Fischer's exact test was used to quantify significance.
